# Diabetes Status and Being Up-to-Date on Colorectal Cancer Screening, 2012 Behavioral Risk Factor Surveillance System

**DOI:** 10.5888/pcd13.150391

**Published:** 2016-02-04

**Authors:** Nancy R. Porter, Jan M. Eberth, Marsha E. Samson, Oralia Garcia-Dominic, Eugene J. Lengerich, Mario Schootman

**Affiliations:** Author Affiliations: Nancy R. Porter, Marsha E. Samson, Arnold School of Public Health, University of South Carolina, Columbia, South Carolina. Oralia Garcia-Dominic, Highmark Blue Shield, Camp Hill, Pennsylvania; College of Medicine, The Pennsylvania State University, Hershey, Pennsylvania; Penn State Hershey Cancer Institute, Hershey, Pennsylvania; College of Health and Human Development, and The Pennsylvania State University, University Park, Pennsylvania. Eugene J. Lengerich, College of Medicine, The Pennsylvania State University, Hershey, Pennsylvania; Penn State Hershey Cancer Institute, Hershey, Pennsylvania; College of Health and Human Development, The Pennsylvania State University, University Park, Pennsylvania. Mario Schootman, Saint Louis University, Saint Louis, Missouri, and Alvin J. Siteman Cancer Center at Barnes-Jewish Hospital and Washington University School of Medicine, Saint Louis, Missouri.

## Abstract

**Introduction:**

Although screening rates for colorectal cancer are increasing, 22 million Americans are not up-to-date with recommendations. People with diabetes are an important and rapidly growing group at increased risk for colorectal cancer. Screening status and predictors of being up-to-date on screening are largely unknown in this population.

**Methods:**

This study used logistic regression modeling and data from the 2012 Behavioral Risk Factor Surveillance System to examine the association between diabetes and colorectal cancer screening predictors with being up-to-date on colorectal cancer screening according to criteria of the US Preventive Services Task Force for adults aged 50 or older. State prevalence rates of up-to-date colorectal cancer screening were also calculated and mapped.

**Results:**

The prevalence of being up-to-date with colorectal cancer screening for all respondents aged 50 or older was 65.6%; for respondents with diabetes, the rate was 69.2%. Respondents with diabetes were 22% more likely to be up-to-date on colorectal cancer screening than those without diabetes. Among those with diabetes, having a routine checkup within the previous year significantly increased the odds of being up-to-date on colorectal cancer screening (odds ratio, 1.90). Other factors such as age, income, education, race/ethnicity, insurance status, and history of cancer were also associated with up-to-date status.

**Conclusion:**

Regardless of diabetes status, people who had a routine checkup within the past year were more likely to be up-to-date than people who had not. Among people with diabetes, the duration between routine checkups may be of greater importance than the frequency of diabetes-related doctor visits. Continued efforts should be made to ensure that routine care visits occur regularly to address the preventive health needs of patients with and patients without diabetes.

## Introduction

Colorectal cancer (CRC) is the second leading cause of cancer-related death in the United States (1) and is positively associated with type 2 diabetes (2) because of shared risk factors (1–3). Biologic mechanisms may also increase CRC risk for people with diabetes (1–3), possibly because increased exposure of colonic mucosa to carcinogens caused by slower bowel transit times increases fecal bile acids associated with blood glucose and triglycerides (1).

Treatment of age-appropriate, screening-detected polyps and early-stage cancer reduces CRC incidence and mortality: the 5-year CRC survival rate is approximately 90% when CRC is found early and treated (4,5). Approximately 22 million Americans are not up-to-date with CRC screening (6). According to the US Preventive Services Task Force (USPSTF) and the American Cancer Society (ACS), a person is up-to-date on CRC screening if he or she has had a fecal occult blood test (FOBT) within the past year, a sigmoidoscopy within the past 5 years and an FOBT within the past 3 years, or a colonoscopy within the past 10 years (4). USPSTF and ACS recommendations differ by age: USPSTF recommends screening for both men and women aged 50 to 75, but ACS does not recommend stopping at age 75 (3,4).

Data on rates of cancer screening by diabetes status are not definitive (7–11). One study found that women with diabetes aged 67 years or older were less likely (odds ratio, 0.79) than same-aged women without diabetes to receive CRC screening (8). This study also found that higher screening rates were associated with increasing numbers of physician visits and diabetes preventive services (8). Other researchers found that people with chronic diseases (12–14) are frequently underscreened (8–12) despite more health care visits.

We examined the association between self-reported diabetes and being up-to-date on CRC screening and predictors of being up-to-date on CRC screening among adults aged 50 or older years overall and by diabetes status.

## Methods

### Survey design

We conducted analyses using 2012 Behavioral Risk Factor Surveillance System (BRFSS) data (15). We limited the study population to respondents aged 50 years or older; respondents younger than 50 years are not asked questions about their CRC screening behavior, because they do not meet the screening age recommended by USPSTF or ACS.

Respondents with a history of colon or rectal cancer were excluded; because they are at increased risk of CRC, the screening guidelines for people of average risk do not apply to them (16). Established in 1984, the BRFSS is the world’s largest continuously conducted health survey system; it is a cross-sectional, random-digit–dial telephone (landline and cell) survey of noninstitutionalized US adults on health-related behaviors, chronic health conditions, and use of preventive services (17). This study was approved by the University of South Carolina’s institutional review board.

### Variables

The outcome of interest was being up-to-date on CRC screening based on the latest USPSTF criteria (4). BRFSS respondents were given a brief description of each screening test and then asked whether they ever had any of them. If they responded yes for any test, they were asked how long it had been since their last one. We used these responses to calculate a composite variable representing a bivariate up-to-date screening status.

Self-reported diabetes status was the main exposure of interest and was measured by using the question “Have you ever been told by a physician that you have diabetes?” We categorized respondents who answered yes as having diabetes, and we excluded women who responded “yes, but told only during pregnancy” from analyses.

A comorbidity score was calculated by using a summary score based on the self-report of 4 conditions (heart attack, angina or coronary heart disease, stroke, or asthma) from a series of 4 questions: “Have you ever been told by a physician that you have [comorbidity]?” Those who responded yes to a comorbidity were given a score of 1 for that question; the comorbidity score had a range of 0 to 4, depending on the number of comorbidities reported.

We categorized respondents who answered no or “no, pre-diabetes or borderline diabetes” as not having diabetes. Both type 1 and type 2 diabetes are included in this question because BRFSS does not ask respondents to make this differentiation. Demographic covariates included age (50–69 y, ≥70 y), sex (female, male), race/ethnicity (white non-Hispanic, nonwhite [including white Hispanic]), marital status (married or one of a couple, not married), education level (high school graduate or less, at least some college) and annual household income (<$35,000, ≥$35,000). Other covariates of interest included body mass index (BMI in kg/m^2^ calculated from self-reported height and weight), general health status, exercise in the past 30 days, health insurance coverage, length of time since the most recent routine checkup, number of visits in the past year for diabetes care, comorbidity score, and history of cancer; these questions have been detailed elsewhere (18). Respondents who reported having diabetes also reported the number of times in the past year they had seen a health professional for their diabetes. Selection of covariates was based on reports in scientific literature (8,11,19,20).

### Statistical analyses

We analyzed data using SAS Version 9.4 (21). We used weighted SAS survey procedures for all analyses because of the complex survey design of BRFSS. We used univariate analyses to determine the relationship of the covariates with the outcome. We used multivariable logistic regression to examine the relationship of diabetes and being up-to-date on CRC screening, adjusting for age, sex, race/ethnicity, income, education, health insurance status, marital status, BMI, physical activity, history of cancer, time since most recent routine checkup, and comorbidity score (Model 1). To determine the final multivariable logistic regression model, we used model selection procedures using manual backward elimination with a cutoff of *P* < .10 for regression coefficients. Variables with *P* > .10 were not included in the final multivariable models. We examined interactions between diabetes and the other covariates; we included interactions significant at *P* < .05 in the multivariable logistic regression model.

We analyzed data on the subset of respondents with diabetes. We used the same model selection procedures for the multivariable logistic regression model for respondents with diabetes (Model 2) that we used in Model 1; we did not examine interactions, because the main variable of interest in interaction testing was diabetes status.

Multivariable logistic regression examined the relationship between covariates and being up-to-date on CRC screening among respondents with diabetes (adjusting for age, sex, income, education, health insurance status, marital status, BMI, physical activity, health status, history of cancer, time since most recent routine checkup, comorbidity score, and number of diabetes-related visits in past year). In a post-hoc analysis, we analyzed whether respondents with diabetes were more likely or less likely than respondents without diabetes to use colonoscopy (or FOBT) for CRC screening. Finally, we developed choropleth maps depicting the state prevalence of adults aged 50 or older being up-to-date on CRC screening using ArcGIS Version 10.1 (22).

## Results

We included data on 258,448 respondents (unweighted) in the analytic sample. Overall, the majority of respondents were women (51.5%), were aged 50 to 69 years (71.9%), were white non-Hispanic (73.8%), had annual household income greater than $35,000 (49.9%), completed some college (54.5%), were married or one of a couple (61.8%), were overweight or obese (68.7%), and had exercised in the past 30 days (71.5%) (Table 1). Approximately 3 of 4 respondents reported having a routine checkup within the past year (78.4%), reported being in good or better health (75.4%), had no comorbidities (80.0%), and reported having some form of health coverage (89.9%); 11.3% reported a history of cancer other than CRC. Approximately 1 of 6 respondents reported having diabetes (18.8%).

**Table 1:** Sociodemographic Characteristics of Adults Aged ≥50, by Diabetes Status and Whether Up-to-Date on Colorectal Cancer Screening, 2012 BRFSS^a^

Variable	Adults Aged ≥50		Adults Aged ≥50 With Diabetes	
	All	Up-to-Date^b^	All	Up-to-Date^b^
**Overall**	NA	65.6	18.8	69.2
**Age, y**				
50–69	71.9	62.8	65.9	67.0
≥70	28.1	73.1	34.1	73.7
**Sex**				
Male	48.5	64.5	50.8	68.9
Female	51.5	66.6	49.2	69.5
**Race**				
Non-Hispanic white	73.8	67.7	63.1	71.4
Nonwhite	26.2	59.6	36.9	65.3
**Annual household income, $**				
<35,000	36.2	57.8	48.3	64.2
≥35,000	49.9	71.1	37.9	75.5
Don’t know or refused to answer	13.9	65.8	13.8	68.5
**Education**				
High school graduate or less	45.5	59.4	55.0	64.8
At least some college	54.5	70.7	45.0	74.5
**Health insurance status**				
Insured	89.9	69.3	91.0	71.8
Uninsured	10.1	32.6	9.0	42.6
**Marital status**				
Married or one of a couple	61.8	68.9	56.7	71.9
Not married	38.2	60.2	43.3	65.6
**Body mass index, kg/m^2^**				
<25.0 (Underweight/normal)	31.3	64.5	16.0	68.3
≥25.0 (Overweight/obese)	68.7	66.5	84.0	69.8
**Exercised in past 30 days**				
No	28.5	60.8	39.5	65.4
Yes	71.5	67.6	60.5	71.6
**Self-rated general health**				
Good or better	75.4	66.5	52.0	71.6
Fair or poor	24.6	63.1	48.0	66.7
**History of cancer other than colorectal cancer**				
No	88.7	63.9	86.8	67.6
Yes	11.3	79.2	13.2	79.5
**Time since most recent routine checkup**				
<1 year	78.4	72.2	88.2	71.7
≥1 year	21.6	42.6	11.8	52.4
**Comorbidity score**				
0	80.0	64.5	60.3	68.0
1	16.1	68.6	23.9	70.1
2	3.0	70.1	11.2	72.5
3	0.8	70.7	3.9	74.7
4	0.1	57.9	0.7	55.6
**Number of diabetes visits in past year**				
Mean (SE)	NA	NA	3.5 (0.05)	3.5 (0.06)
0-2	NA	NA	31.8	69.7
3-6	NA	NA	47.2	70.6
≥7	NA	NA	21.0	63.4

Abbreviations: BRFSS, Behavioral Risk Factor Surveillance System (15); NA, not applicable; SE, standard error.

a All values are percentages unless otherwise indicated.

b Defined as having a fecal occult blood test within 1 year, sigmoidoscopy within 5 years and a fecal occult blood test within 3 years, or colonoscopy within 10 years.

The characteristics of respondents with diabetes were similar to those of the overall population for sex, income, education, and marital status. However, respondents with diabetes were more likely to be older (≥70 y, 34.1% vs 28.1%), nonwhite (36.9% vs 26.2%), and overweight or obese (84.0% vs 68.7%); and more likely to report not having exercised in the past 30 days (39.5% vs 28.5%), fair to poor health (48.0% vs 24.6%), and having a routine checkup within the past year (88.2% vs 78.4%).The average number of doctor visits for diabetes care in the past year was 3.5 visits; 31.8% of respondents had 0, 1, or 2 diabetes care visits, and 47.2% had 3 to 6 diabetes care visits. Comorbidities were more prevalent among respondents with diabetes than among those in the overall sample population (Table 1).

The prevalence of reported up-to-date CRC screening was 65.6% for all respondents and 69.2% among respondents with diabetes (Table 1). The prevalence of being up-to-date differed by state; we found higher rates in the Northeast than in Alaska, the Southwest, and the Midwest (Figure). Alaska had the lowest overall prevalence of being up-to-date (56.3%), whereas Massachusetts had the highest overall prevalence (75.5%). In some states, the prevalence of being up-to-date was higher among respondents with diabetes than among those in the overall sample. For example, the prevalence of being up-to-date in Alaska’s overall population was 56.3% and among respondents with diabetes, it was 72.5%. This difference was nearly 10 percentage points in Delaware, Idaho, Louisiana, and Nevada. In contrast, the prevalence of being up-to-date was lower among respondents with diabetes than among those in the overall sample in Connecticut, the District of Columbia, and South Dakota (Figure).

**Figure:**
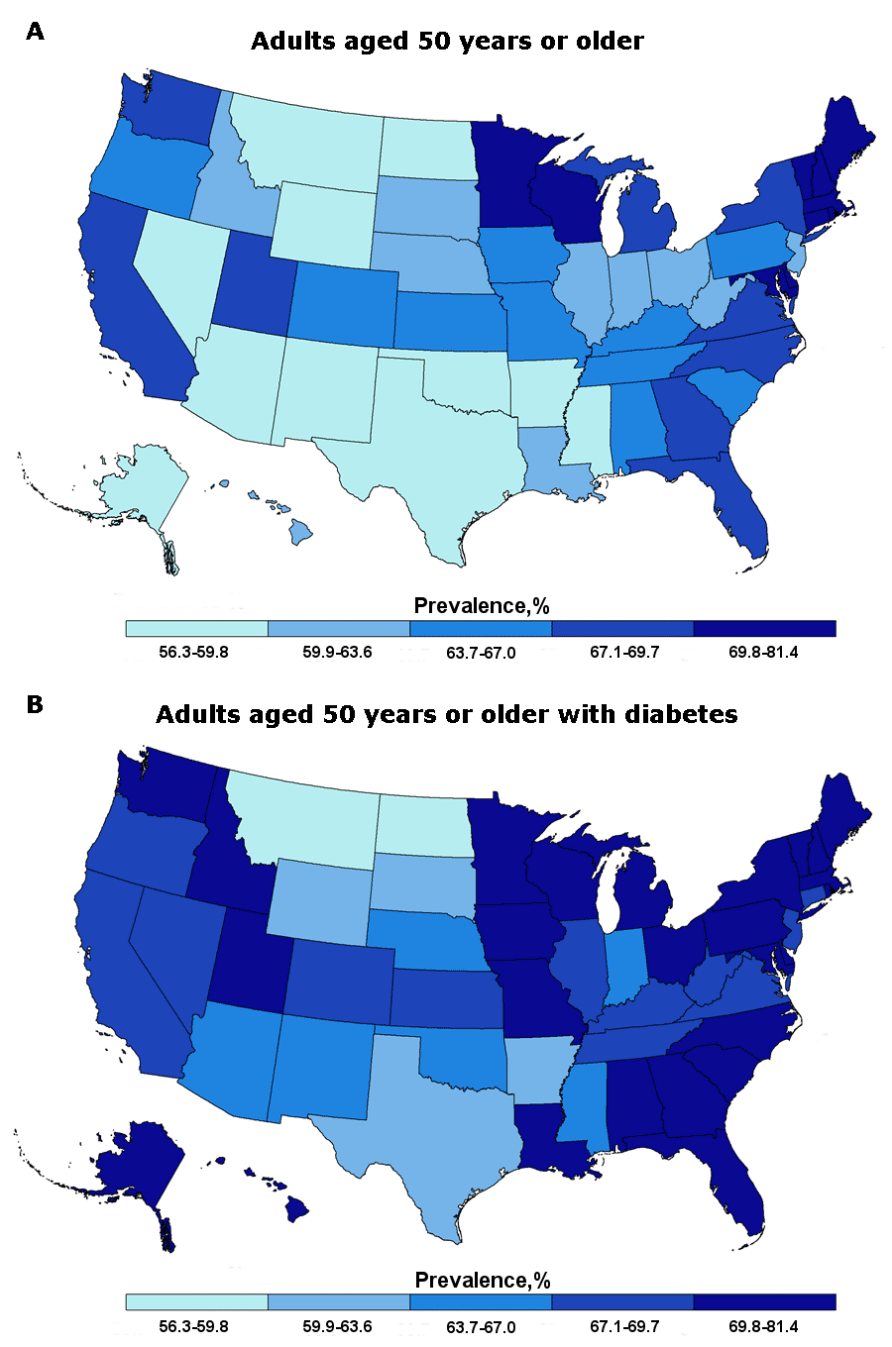
Prevalence of up-to-date colorectal cancer screening among A) adults aged 50 years or older and B) adults aged 50 years or older with diabetes, Behavioral Risk Factor Surveillance System (BRFSS), 2012. **Table:** 

State	All Adults Aged 50 or Older, Up-to-Date, %	Adults Aged 50 or Older With Diabetes, Up-to-Date, %
Alabama	65.5	70.8
Alaska	56.3	72.5
Arizona	59.4	66.2
Arkansas	58.1	61.3
California	67.5	67.6
Colorado	65.2	69.2
Connecticut	72.3	69.2
Delaware	72.1	81.4
District of Columbia	68.2	67.5
Florida	67.1	71.4
Georgia	67.6	71.7
Hawaii	62.9	70.8
Idaho	61.2	71.1
Illinois	61.9	69.5
Indiana	60.4	64.5
Iowa	66.6	70.8
Kansas	65.6	69.2
Kentucky	63.9	69.0
Louisiana	61.8	70.0
Maine	72.9	76.7
Maryland	70.3	73.3
Massachusetts	75.5	76.5
Michigan	69.1	72.4
Minnesota	70.1	72.0
Mississippi	59.1	66.9
Missouri	64.5	72.0
Montana	57.0	57.7
Nebraska	61.6	64.9
Nevada	59.2	67.6
New Hampshire	74.6	78.7
New Jersey	63.0	67.7
New Mexico	58.7	65.7
New York	69.7	76.6
North Carolina	69.3	73.3
North Dakota	58.8	59.1
Ohio	63.6	71.0
Oklahoma	59.8	64.9
Oregon	64.9	68.9
Pennsylvania	67.0	70.4
Rhode Island	73.0	74.7
South Carolina	65.2	69.9
South Dakota	63.1	60.9
Tennessee	65.4	69.5
Texas	59.6	62.7
Utah	67.8	69.9
Vermont	70.9	77.1
Virginia	67.8	69.1
Washington	67.4	70.7
West Virginia	63.3	69.3
Wisconsin	71.9	74.0
Wyoming	57.0	63.2

For the overall population, univariate analyses showed that all variables were strongly associated with being up-to-date on CRC screening (*P* < .05). Respondents with diabetes had approximately 22% greater odds of being up-to-date than respondents without diabetes (Table 2). The multivariable logistic regression model (Model 1) showed that the odds of being up-to-date on CRC screening varied by diabetes status and by time since most recent routine checkup. Among those who had not had a routine checkup within the past year, respondents with diabetes were more likely to be up-to-date than respondents without diabetes (odds ratio [OR], 1.70). Respondents with diabetes who had a routine checkup within the past year were nearly twice as likely to be up-to-date as respondents with diabetes who had not had a routine checkup within the past year (OR, 1.90). In the general population, other predictors of being up-to-date on CRC screening included older age, higher income, higher BMI, being physically active, having 1 to 3 comorbidities (vs none), female sex, and history of cancer (Table 2). A race/ethnicity other than non-Hispanic white, being uninsured, having fair or poor health, and single marital status significantly decreased the odds of being up-to-date on screening. We found similar associations in the model for respondents with diabetes (Model 2). The number of diabetes-related visits in the past year was not significantly associated with up-to-date CRC screening after we adjusted for other covariates. Among respondents with diabetes, having 4 self-reported comorbidities was associated with decreased odds of screening, whereas having 1 to 3 self-reported comorbidities increased the likelihood of being up-to-date.

**Table 2:** Odds Ratios (95% Confidence Intervals) of Being Up-to-Date^a^ on Colorectal Cancer Screening, Adults Aged ≥50, by Diabetes Status, 2012 BRFSS

Variable	Adults Aged ≥50		Adults Aged ≥50 With Diabetes	
	Univariate Analysis	Multivariable Model 1	Univariate Analysis	Multivariable Model 2
**Diabetes**				
No	1 [Ref]	NA	NA	NA
Yes	1.22 (1.16–1.27)	NA	NA	NA
**Age, y**				
50–69	1 [Ref]	1 [Ref]	1 [Ref]	1 [Ref]
≥70	1.61 (1.55–1.67)	1.40 (1.34–1.46)	1.38 (1.26–1.52)	1.36 (1.23–1.50)
**Sex**				
Male	1 [Ref]	1 [Ref]	1 [Ref]	1 [Ref]
Female	1.10 (1.06–1.14)	1.12 (1.07–1.16)	1.03 (0.94–1.12)	1.13 (1.02–1.25)
**Race**				
Non-Hispanic white	1 [Ref]	1 [Ref]	1 [Ref]	NI
Nonwhite	0.70 (0.67–0.74)	0.89 (0.85–0.94)	0.75 (0.68–0.83)	NI
**Annual household income, $**				
<35,000	1 [Ref]	1 [Ref]	1 [Ref]	1 [Ref]
≥35,000	1.79 (1.73–1.86)	1.32 (1.26–1.38)	1.72 (1.57–1.89)	1.36 (1.20–1.54)
Don’t know or refused to answer	1.40 (1.33–1.48)	1.12 (1.05–1.19)	1.21 (1.05–1.39)	1.09 (0.96–1.25)
**Education**				
High school graduate or less	1 [Ref]	1 [Ref]	1 [Ref]	1 [Ref]
At least some college	1.65 (1.59–1.70)	1.44 (1.39–1.50)	1.58 (1.45–1.72)	1.39 (1.26–1.54)
**Health insurance status**				
Insured	1 [Ref]	1 [Ref]	1 [Ref]	1 [Ref]
Uninsured	0.21 (0.20–0.23)	0.40 (0.37–0.43)	0.29 (0.25–0.34)	0.46 (0.38–0.56)
**Marital status**				
Married or one of a couple	1 [Ref]	1 [Ref]	1 [Ref]	1 [Ref]
Not married	0.68 (0.66–0.70)	0.75 (0.72–0.79)	0.74 (0.68–0.81)	0.80 (0.72–0.88)
**Body mass index, kg/m^2^**				
<25.0 (Underweight/normal)	1 [Ref]	1 [Ref]	1 [Ref]	1 [Ref]
≥25.0 (Overweight/obese)	1.09 (1.05–1.13)	1.12 (1.08–1.17)	1.07 (0.94–1.22)	1.25 (1.09–1.43)
**Exercised in past 30 days**				
No	1 [Ref]	1 [Ref]	1 [Ref]	1 [Ref]
Yes	1.35 (1.30–1.40)	1.25 (1.19–1.30)	1.34 (1.23–1.46)	1.25 (1.13–1.38)
**General health**				
Good or better	1 [Ref]	NI	1 [Ref]	1 [Ref]
Fair or poor	0.86 (0.83–0.90)	NI	0.79 (0.73–0.87)	0.87 (0.78- 0.97)
**History of cancer other than colorectal cancer**				
No	1 [Ref]	1 [Ref]	1 [Ref]	1 [Ref]
Yes	2.15 (2.04–2.28)	1.85 (1.74–1.97)	1.86 (1.63–2.12)	1.65 (1.41–1.94)
**Time since most recent routine checkup**				
<1 year	1 [Ref]	1 [Ref]	1 [Ref]	1 [Ref]
≥1 year	0.29 (0.27–0.30)	NA	0.44 (0.38–0.50)	0.60 (0.50–0.69)
**Comorbidity score**				
0	1 [Ref]	1 [Ref]	1 [Ref]	1 [Ref]
1	1.20 (1.15–1.25)	1.13 (1.08–1.19)	1.10 (1.00–1.22)	1.13 (1.01–1.27)
2	1.29 (1.20–1.38)	1.18 (1.09–1.29)	1.24 (1.07–1.42)	1.34 (1.14–1.57)
3	1.33 (1.14–1.55)	1.25 (1.05–1.49)	1.38 (1.09–1.75)	1.41 (1.08–1.86)
4	0.76 (0.54–1.06)	0.79 (0.52–1.20)	0.59 (0.36–0.95)	1.04 (0.60–1.79)
**Number of diabetes visits in past year**				
0–2	NA	NA	1 [Ref]	1 [Ref]
3–6	NA	NA	1.05 (0.94–1.16)	1.07 (0.96–1.19)
≥7	NA	NA	0.75 (0.66–0.86)	0.89 (0.78–1.02)
**Diabetes × time since most recent routine checkup**				
≥1 Year since most recent checkup: has diabetes vs does not have diabetes	NA	1.70 (1.48–1.96)	NA	NA
Has diabetes: <1 y vs ≥1 y since most recent checkup	NA	1.90 (1.65–2.19)	NA	NA

Abbreviations: BRFSS, Behavioral Risk Factor Surveillance System (15); NA, not applicable; NI, not included in model as a result of model selection procedure; Ref, reference.

a Defined as having a fecal occult blood test within 1 year, sigmoidoscopy within 5 years and a fecal occult test within 3 years, or colonoscopy within 10 years.

In the post-hoc analysis, we found that compared with the general population, respondents with diabetes were slightly less likely to use colonoscopy (83.3% vs 84.2%) and more likely to use FOBT (5.7% vs 4.2%). Most of the general US population, as well as respondents with diabetes, used colonoscopy to screen for CRC.

## Discussion

The prevalence of BRFSS respondents in 2012 who were up-to-date on CRC screening was higher for those with diabetes than for those in the general population nationally and in nearly all states. Regardless of diabetes status, respondents who had a routine checkup within the past year were more likely to be up-to-date with CRC screening than respondents who had not. This finding emphasizes the importance of a routine checkup for preventive health care. However, among those who had not had a routine checkup within the past year, respondents with diabetes were more likely to be up-to-date than respondents without diabetes. Thus, people with diabetes seem to be more likely to be up-to-date on their CRC screening even if they have not had a recent routine checkup. Our results agree with the results of studies reporting that people with diabetes are as likely as, or more likely than, people without diabetes to receive CRC screening (7,8,23,24). The increased probability of being up-to-date with CRC screening guidelines may be due to increased contact between people with diabetes and the medical system. Among people with diabetes, the duration between routine checkups may be a more important factor than the frequency of diabetes-related doctor visits. We found that when diabetes-related visits in the past year exceeded 7 visits, the patient had significantly decreased odds of being up-to-date. However, this association was not significant after we adjusted for other covariates. This finding is consistent with other findings showing that people with chronic diseases are underscreened, despite more health care visits (7–9,19). A reason for these findings may be that preventive services are discussed during routine visits, rather than during diabetes-related visits; the diabetes specialist may defer preventive services such as cancer screenings to the primary care provider, or if the patient sees a primary care physician for his or her diabetes care, the physician might wait until the patient’s next routine checkup to discuss CRC screening. The recent adoption of the patient-centered medical home model in improving the quality of diabetes care and comprehensively addressing the needs of chronically ill patients may have influenced our findings (25). Additionally, in the era of widespread use of electronic medical records (EMRs), primary care physicians and diabetes care specialists could rely on EMR-based prompts to alert them when patients are eligible (or due) for screening.

The differences in up-to-date status across states may be due to various factors, including, but not limited to, compositional effects (eg, population age distribution and racial/ethnic mix), differences in geographic access to CRC screening providers or facilities (26), primary care shortages (27), area poverty (28–30), or state factors, such as policies requiring CRC screening coverage (31). Data sets with more geographic granularity and statistical power may be able to tease out the effects of such factors. With full implementation of the Affordable Care Act, state differences may also be mitigated, as more people enroll in health insurance plans that cover CRC screening as a preventive service without patient cost-sharing.

This study used data from the BRFSS, which has strengths and limitations. BRFSS respondents self-reported their diabetes status, and blood glucose levels were not measured, which may have led to an overreporting of diabetes prevalence. However, using data from the National Health and Nutrition Examination Survey, one study reported a diabetes prevalence of 17.5% among people 45 to 64 years and a prevalence of 33.0% among those 65 or older, which is consistent with the prevalence of 18.8% among people 50 years or older found in our study (32). In addition, studies found that self-reported diabetes and sociodemographic data in the BRFSS are valid and reliable (33,34). A study using data from 2007 found only minor differences in self-reported diabetes status between the BRFSS and the 2 most commonly used population-based health surveys in the United States, the National Health Interview Survey (NHIS) and the National Health and Nutrition Examination Survey (NHANES) (35). Thus, despite this potential shortcoming, the BRFSS is considered one of the best tools to monitor leading health indicators using population-based self-reported data (35). Future studies should examine the relationship between up-to-date CRC screening status and prediabetes and the relationship between motivation and intent to get screened for CRC among people with diabetes and people without diabetes, as well as potential external influences on CRC screening adherence among people with diabetes (eg, physician recommendation, geographic access to colonoscopy providers, state screening initiatives or policies). Because studies documented nonstationarity in predictors of CRC screening adherence in the general population across US states (36,37), further research is also needed to examine differences in predictors of CRC screening adherence among people with diabetes.

The prevalence of adults aged 50 or older who are up-to-date on their CRC screening was 65.6%, well below the target of 80% set by the National Colorectal Cancer Roundtable (38). Because people with diabetes are at an increased risk of developing CRC (1), it is encouraging to observe higher screening rates for the population with diabetes (69.2% up-to-date nationally, and near 80% in Delaware, New Hampshire, and Maine). Screening programs need to continue targeting this population, especially in Alaska, the Southwest, and Midwest, where the prevalence of people up-to-date on CRC screening was lower than average. These study findings may help inform health professionals designing and implementing programs aimed at improving and maintaining high rates of CRC screening uptake among people with diabetes who are of the recommended age. Results from this study show that among people with diabetes, having a routine checkup within the past year is a strong predictor of being up-to-date; however, the number of diabetes-related visits in the past year did not predict being up-to-date. These findings suggest that diabetes-related visits may represent a missed opportunity to discuss CRC screening, especially given the greater risk of CRC among people with diabetes than among people without diabetes.
